# Targeted Delivery of Immunotoxin by Antibody to Ganglioside GD3: A Novel Drug Delivery Route for Tumor Cells

**DOI:** 10.1371/journal.pone.0055304

**Published:** 2013-01-31

**Authors:** Vanina Torres Demichelis, Aldo A. Vilcaes, Ramiro Iglesias-Bartolomé, Fernando M. Ruggiero, Jose L. Daniotti

**Affiliations:** Centro de Investigaciones en Química Biológica de Córdoba (CIQUIBIC, UNC-CONICET), Departamento de Química Biológica, Facultad de Ciencias Químicas, Universidad Nacional de Córdoba, Córdoba, Argentina; Ospedale Pediatrico Bambino Gesu’, Italy

## Abstract

Gangliosides are sialic acid-containing glycolipids expressed on plasma membranes from nearly all vertebrate cells. The expression of ganglioside GD3, which plays essential roles in normal brain development, decreases in adults but is up regulated in neuroectodermal and epithelial derived cancers. R24 antibody, directed against ganglioside GD3, is a validated tumor target which is specifically endocytosed and accumulated in endosomes. Here, we exploit the internalization feature of the R24 antibody for the selective delivery of saporin, a ribosome-inactivating protein, to GD3-expressing cells [human (SK-Mel-28) and mouse (B16) melanoma cells and Chinese hamster ovary (CHO)-K1 cells]. This immunotoxin showed a specific cytotoxicity on tumor cells grew on 2D monolayers, which was further evident by the lack of any effect on GD3-negative cells. To estimate the potential antitumor activity of R24-saporin complex, we also evaluated the effect of the immunotoxin on the clonogenic growth of SK-Mel-28 and CHO-K1^GD3+^ cells cultured in attachment-free conditions. A drastic growth inhibition (>80–90%) of the cell colonies was reached after 3 days of immunotoxin treatment. By the contrary, colonies continue to growth at the same concentration of the immuntoxin, but in the absence of R24 antibody, or in the absence of both immunotoxin and R24, undoubtedly indicating the specificity of the effect observed. Thus, the ganglioside GD3 emerge as a novel and attractive class of cell surface molecule for targeted delivery of cytotoxic agents and, therefore, provides a rationale for future therapeutic intervention in cancer.

## Introduction

Gangliosides are a heterogeneous family of sialic acid-containing glycosphingolipids present on plasma membranes, where they participate in cell-surface events such as modulation of growth factor receptors and cell-to-cell and cell-to matrix interactions [1]. Aberrant glycosilation occurs in essentially all types of experimental and human cancers, and many glycosil epitopes constitute tumor-associated antigens [2]. The expression of non-normal glycosil epitopes is believed to affect tumor progression, promoting or inhibiting it [2]. Malignant transformation of cells, especially those of neuroectodermal origin (like melanoma and neuroblastoma), often result in elevated expression of gangliosides such us GM2, GD2, GD3 and 9-O-acetyl-GD3 [3], [4].

Antibody-based cancer immunotherapies use antibody dependent cellular cytotoxicity and complement-dependent cytotoxicity, or enhance natural effects of antibodies by arming these with radioisotopes, toxins or drugs. Thus, several types of targeted therapy need that the antibody remains at the cell surface to mediate cytotoxicity, but other therapies need internalization and drug release into the cell [5]. Anti-ganglioside antibodies are available, like anti-GD2 for neuroblastoma [6] and anti-GD3 for melanoma [7]. Particularly, mouse monoclonal R24 antibody (IgG3), directed against ganglioside GD3, is a validated tumor targeting agent that shows strong cell surface reactivity with a range of human melanoma cell lines and other epithelial cancer tumor cells [7].

In a previous study, we demonstrated that the R24 antibody is rapidly endocytosed after binding to the disialo ganglioside GD3, sorted to early endosomes, latter accumulated in the recycling endosome and finally transported back to the plasma membrane [8]. Its rapid internalization in cells precludes its use as a “naked” therapeutic because when internalized it cannot link to pathways of complement- and cellular- dependent anticancer activity. However, it would be possible to exploit the internalization feature for the selective delivery of cytotoxic agents to GD3-expressing cancer cells. In this study, we take advantage of the internalization feature of R24 antibody for selective delivery of saporin, a ribosome-inactivating protein, to GD3-expressing cells. First, the ablation cell strategy was successfully established in Chinese hamster ovary (CHO)-K1 cells and latter demonstrated to be effective both in human (SK-Mel-28) and mouse (B16) melanoma cells. We propose that chemical coupling of antibodies to gangliosides with chemotherapeutics would allow the intracellular delivery of cytotoxic agents and, therefore, provides a rationale for future therapeutic intervention in cancer.

## Materials and Methods

### Inhibition of In Vitro Cell Proliferation

The following cells were used: wild-type CHO-K1 (CHO-K1^wt^) cells (ATCC, Manassas, VA, USA); CHO-K1 clone 2 (CHO-K1^GD3+^), a stable CMP-NeuAc:GM3α2,8-sialyltransferase (Sial-T2, tagged at the C-terminus with the nanopeptide epítope of the viral hemagglutinin) transfectant expressing the ganglioside GD3 [9], [10]; and the SK-Mel 28 and B16 melanoma cell lines (ATCC). Cells were grown and maintained at 37°C in 5% CO_2_ in Dulbecco’s modified Eagle’s medium (DMEM) supplemented with 10% fetal bovine serum (FBS) and antibiotics. 4500–5000 cells were cultured at 37°C in 96-well plates at the indicated concentration of monoclonal antibody to GD3 (R24 antibody, hybridoma ATCC N° HB-8445) plus goat antibody to mouse IgG or saporin conjugated goat antibody anti mouse IgG secondary antibody (Advance Targeting Systems, San Diego, CA, USA). R24 and secondary antibody complexes were first incubated in an eppendorf and then incorporated into the culture medium. Cell viability was monitored at 72 h employing 3-(4,5 dimethylthiazol-2-yl)-2,5 diphenyltetrazolium bromide (MTT) metabolic reduction. Absorbance was measured at 595 nm using a multiplate reader. Results were analyzed by ANOVA followed by Tukey’s multiple comparison test to determine if significant differences existed between groups (*p*<0.05). Results are given as means±S.E.

### Generation of Stably Transfected Mouse B16 Melanoma Cell Clone Expressing Sial-T2

Wild-type B16 mouse melanoma cells (B16^wt^) grown in DMEM medium-10% FBS at 37°C in 5% CO_2_ were transfected with 1 µg/dish pCEFL-Sial-T2-HA using Lipofectamine (Invitrogen, Carlsbad, CA, USA) [10]. After 24 h of expression, the cells were cultured in DMEM containing 10% FBS and 1 mg/ml geneticin (G418). Colonies of stable transfectants were screened for GD3 ganglioside expression and GD3-positive cells were isolated by cell sorter procedure. B16^GD3+^ cells were maintained in 0.5 mg/ml G418.

### Soft Agar Colony Assay

This method was carried out essentially as described [11]. 24-well plates were coated with 0.3 ml of 0.5% agar (Sigma-Aldrich, St. Louis, MO, USA) in DMEM supplemented with 20% FBS. For each dish, 50–100 cells were suspended in 0.3 ml medium with 20% FBS, mixed with 1 ml of 0.375% soft agar in DMEM containing 20% FBS, and dispensed into the well plates. Cells were incubated at 37°C in a hummed atmosphere of 5% CO_2_ until cell colonies appeared.

### Cell Labeling and Internalization Assays

Cells grown on coverslips were incubated on ice for 20 min to inhibit intracellular transport. Then, cells were incubated on ice for 45 min with R24 antibody (1∶100 dilution) in order to label GD3 ganglioside expressed on the cell surface. Afterwards, cells were washed three times with cold PBS, transferred to 37°C with fresh prewarmed complete DMEM to allow antibody internalization for 30 min and finally fixed in 4% paraformaldehyde in PBS for 20 min at 4°C. Then, cells were washed twice with PBS and permeabilized with 0.1% Triton X-100/200 mM glycine in PBS for 10 min. Next, cells were washed with PBS and exposed to secondary antibody for 90 min at 37°C. Secondary antibody was Alexa Fluor^488^-conjugated goat anti-mouse IgG (Molecular Probes, Eugene, OR, USA) diluted 1∶1000. After final washes with PBS, cells were mounted in FluorSave reagent (Calbiochem, EMD Biosciences, La Jolla, CA, USA).

### Confocal Immunofluorescence Microscopy

Confocal images were collected using a Carl Zeiss LSM5 Pascal laser-scanning confocal microscope (Carl Zeiss, Jena, Germany) equipped with an argon/helium/neon laser and a 63×1.4 numerical aperture, oil immersion objective (Zeiss Plan-Apochromat) or with an Olympus FluoView™ FV1000 confocal microscope equipped with an argon/helium/neon laser and a 63×1.42 numerical aperture, oil immersion objective. Single confocal sections of 0.7 µm were taken parallel to the coverslip (*xy* sections). Images were acquired and processed with the Zeiss LSM image software or Olympus FluoView FV10-ASW software. Final images were compiled with Adobe Photoshop CS. The fluorescence micrographs shown in this manuscript are representative of at least three independent experiments.

## Results

### Binding and Specific Internalization of R24 Antibody in GD3-Expressing Cells

**Figure 1:**
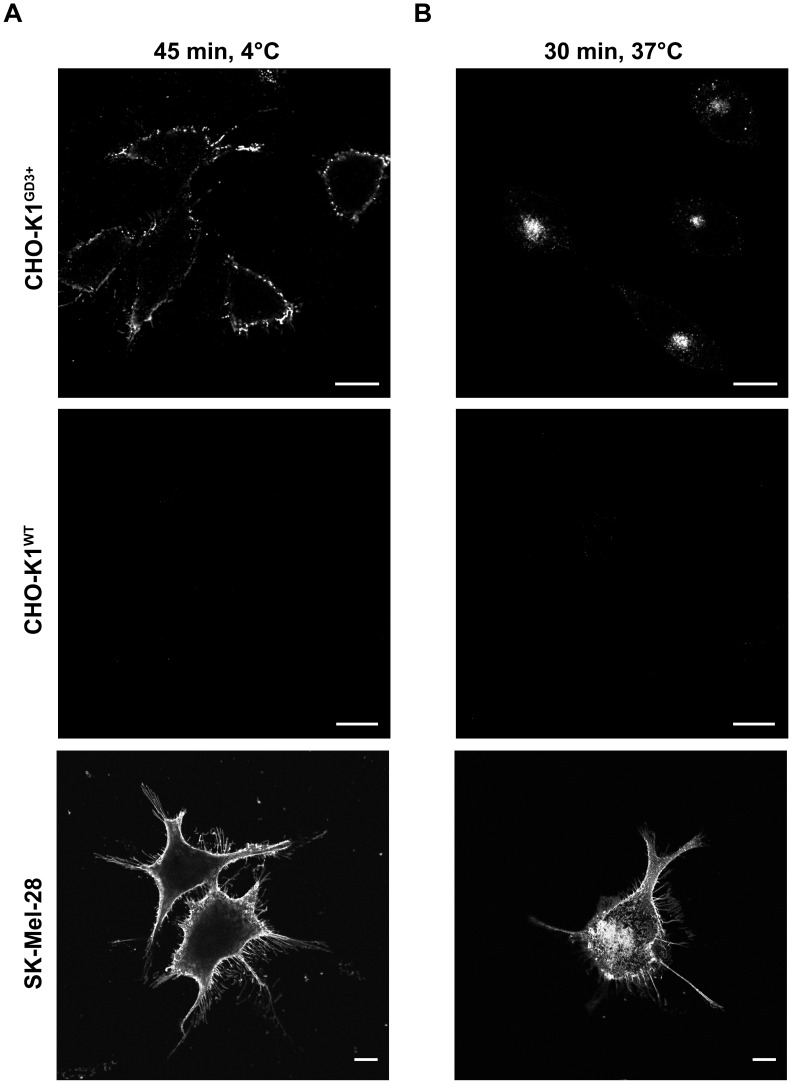
R24 antibody is specifically internalized in GD3-expressing cells. A ) CHO-K1^GD3+^, CHO-K1^WT^ (GD3-) and SK-Mel-28 cells grown on coverslips were incubated at 4°C to inhibit intracellular transport, then with R24 antibody for 45 min at 4°C, washed and fixed. R24 antibody was detected by using goat anti-mouse IgG conjugated with Alexa Fluor^488^ (45 min, 4°C; left panels). **B**) Cells were incubated with R24 for 45 min at 4°C and after washing temperature was shifted to 37°C for 30 min to allow the endocytosis of the complex GD3-R24. Then, cells were fixed and R24 antibody detection was carried out as indicated in **A** (30 min, 37°C; right panel). Single confocal sections were taken every 0.8 µm parallel to the coverslip. The fluorescence micrographs shown are representative of three independent experiments. Scale bar: 10 µm.

Parental CHO-K1 cells only express the ganglioside GM3. CHO-K1 cells stably transfected with the cDNA encoding Sial-T2 (clone 2, CHO-K1^GD3+^ cells) synthesize mostly GD3 [9], [10]. As shown in Figure 1, binding of R24 antibody to live CHO-K1^GD3+^ cells at 4°C had a plasma membrane punctate distribution. After 30 min at 37°C, the internalized R24 antibody became more concentrated in the perinuclear region, which was previously identified as recycling endosomes [8]. Similar binding and internalization of R24 antibody bound to GD3 was also observed in human SK-Mel-28 melanoma cells, which mainly express the ganglioside GD3 and GM3 (Fig. 1). Endocytosis of R24 is specifically mediated by GD3, as wild-type CHO-K1 cells did not bind and internalize R24 (Fig. 1). Thus, the ganglioside GD3 become an attractive cell surface molecule to be targeted in antibody-mediated intracellular delivery of cytotoxic agents in GD3-expressing tumor cells.

### R24-Targeted Immunotoxin Shows Specific Cytotoxicity on Epithelial and Melanoma Cells

**Figure 2:**
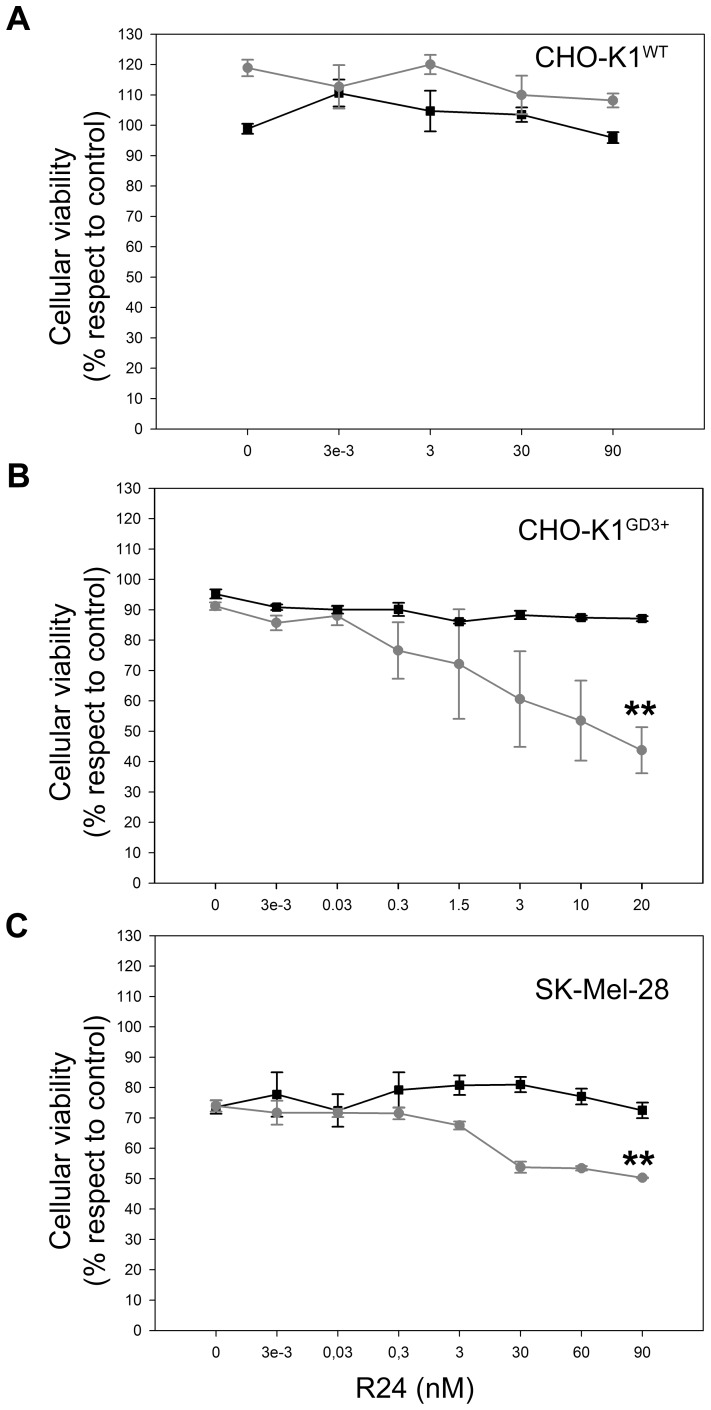
Selective cytotoxicity of R24-targeted immunotoxin on epithelial and melanoma cells. CHO-K1^WT^ (GD3−) (**A**), CHO-K1^GD3+^ (**B**) and SK-Mel-28 (**C**) cells were cultured at 37°C for 72 h in 96-well plates and treated with the indicated concentration of monoclonal antibody to GD3 (R24 antibody) in combination with secondary antibody: goat antibody to mouse IgG (squares, black lines) or saporin conjugated goat antibody to mouse IgG (circles, grey lines). As negative control (100% viability), CHO-K1^WT^, CHO-K1^GD3+^ and SK-Mel-28 cells were incubated only with culture medium. The concentration of the secondary antibodies was as follows: 0.95 nM for CHO-K1^wt^ and CHO-K1^GD3+^ and 9.5 nM for SK-Mel-28. Cell viability was determined using the colorimetric MTT metabolic activity assay. Absorbance was measured at 595 nm using a multiplate reader. Results were analyzed by ANOVA followed by Tukey’s multiple comparison test. Results are given as means±S.E. The relative cell viability (%) was expressed as a percentage relative to the untreated control cells. Note that R24-targeted saporin selectively kill epithelial and melanoma cells (**p<0.001, respect to control condition). CHO-K1^GD3+^ (clone 2) shows some cell-to-cell variability in the expression of GD3 in non-synchronized cultures, which is more evident after several passages. The differences in GD3 expression levels could contribute to the variability of the observations on cells exposed to R24/Saporin-Ab.

To evaluate the possibility mentioned in the last paragraph, we tested whether the antibody to GD3 is able to selectively deliver the toxin saporin into GD3-expressing cells. As a first approach, we used a dual system in which a secondary antibody coupled to saporin is bound to the mouse antibody to GD3 R24. We demonstrated that the intracellular fate and timing of internalization of both R24 and R24-saporin-Ab complex are essentially the same (Figure S1). Initial titration of the R24/Saporin-Ab at 1∶1 molar ratio in CHO-K1^GD3+^ indicated that an R24 concentration of 3 pM was sufficient for initial testing the ability of antibody R24 to deliver saporin into the cells and to induce cell death in a time-dependent process (results not shown). In order to determinate the optimum ratio of Saporin-Ab to the anti GD3 monoclonal antibody (R24) in CHO-K1^GD3+^ cells, the concentration of the secondary antibody-saporin was maintained at 0.95 nM and the primary antibody was varied from 0.003 pM to 20 nM. After 72 h of incubation with R24 at 20 nM, more than 50% of CHO-K1 cells stably expressing GD3 were killed (Fig. 2B). We discard the possibility of selection of GD3 negative cells after 72 h of saporin exposure since the expression GD3 in residual cells was similar to that observed in control cells (Figure S2). As also indicated in Figure 2B, primary antibody R24 in the absence of Saporin-Ab had no a significant effect (<10%) on cell growth. In addition, the complex consisting of control IgG (mouse IgG anti c-Myc) and Saporin-Ab had no effect even after 72 h of treatment (results nor shown). The specificity of R24 antibody for delivering saporin was also determined using wild-type CHO-K1 cells which do not express GD3 and express only GM3 (Fig. 2A). Clearly, this cell line was not affected under the identical culture conditions, even at higher doses of R24 antibody.

**Figure 3:**
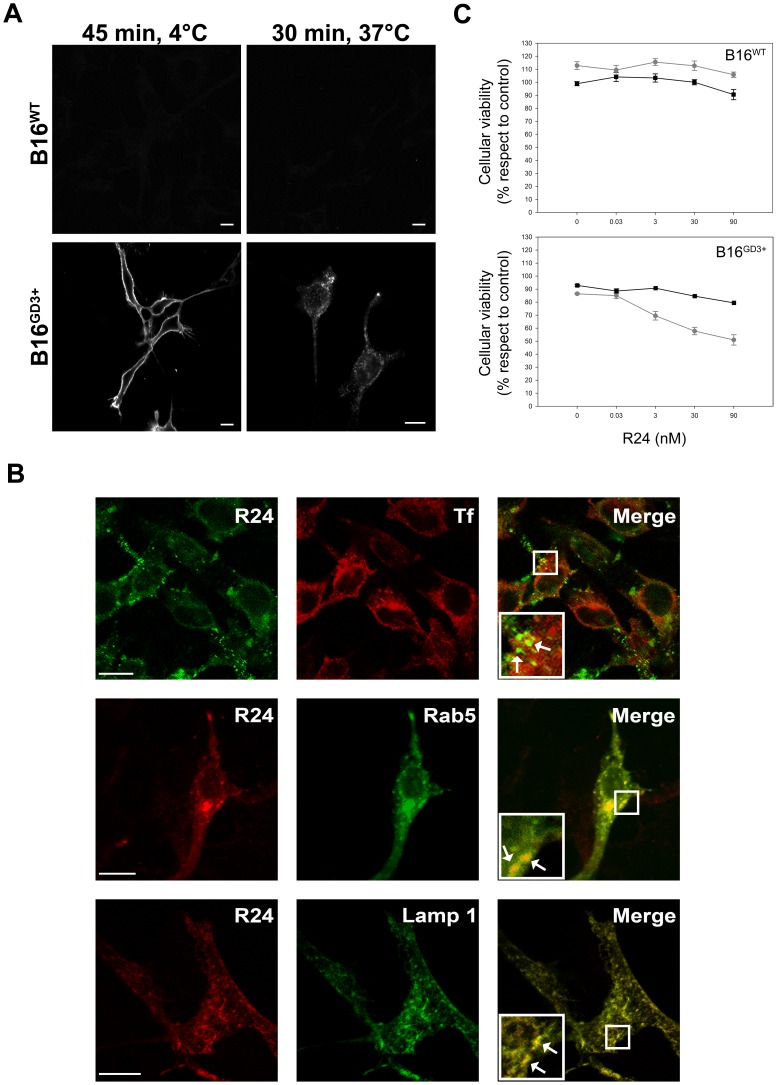
Selective cytotoxicity of R24-targeted immunotoxin on mouse B16^GD3+^ melanoma cells. A ) Wild-type B16 cells (B16^wt^) and B16 cells genetically modified to express GD3 (B16^GD3+^) grown on coverslips were incubated at 4°C to inhibit intracellular transport, then with R24 antibody for 45 min at 4°C, washed and fixed. R24 antibody was detected by using anti-mouse IgG conjugated with Alexa Fluor^488^ (45 min, 4°C; left panels). Cells were incubated with R24 for 45 min at 4°C and after washing temperature was shifted to 37°C for 30 min to allow the endocytosis of the complex GD3-R24. Then, cells were fixed and R24 antibody detection was carried out as indicated above (30 min, 37°C; right panels). **B**) B16^GD3+^ cells transiently expressing Rab5-GFP or Lamp1-GFP were incubated with R24 for 45 min at 4°C. After washing, temperature was shifted to 37°C for 30 min to allow the endocytosis of the complex GD3-R24. R24 antibody was detected by using anti-mouse IgG conjugated with Alexa Fluor^543^. Expression of Rab5 and Lamp1 was detected by the intrinsic fluorescence of GFP. In another set of experiments, uptake of Alexa Fluor^647^-transferrin (Tf) was monitored simultaneously with R24 endocytosis. In this case, R24 antibody was detected by using anti-mouse IgG conjugated with Alexa Fluor^488^. Insets in merge panels (right column) show details at higher magnifications. In all experimental conditions, single confocal sections were taken every 0.8 µm parallel to the coverslip. The fluorescence micrographs shown are representative of three independent experiments. Scale bar: 10 µm. **C**) B16^wt^ and B16^GD3+^ cells were cultured at 37°C for 72 h in 96-well plates at the indicated concentration of monoclonal antibody to GD3 (R24 antibody) in combination with goat antibody to mouse IgG (squares, black lines) or saporin conjugated goat antibody anti mouse IgG secondary antibody (circles, grey lines). The concentration of the secondary antibodies was 0.95 nM. As negative control (100% viability), B16^GD3+^ cells were incubated only with culture medium. Cell viability was determined using the colorimetric MTT metabolic activity assay. Absorbance was measured at 595 nm using a multiplate reader. Results were analyzed by ANOVA followed by Tukey’s multiple comparison test. Results are given as means±S.E. The relative cell viability (%) was expressed as a percentage relative to the untreated control cells. Note that R24-targeted saporin selectively kills B16^GD3+^ melanoma cells (** p<0.001, respect to control condition).

Next, we attempted to evaluate the effect of R24-mediated intracellular delivery of saporin in human SK-Mel-28 melanoma cells, which endogenously express the ganglioside GD3 [8]. As observed in CHO-K1^GD3+^ cells, R24/Saporin-Ab complex killed between 25 and 33% of the cells at 60–90 nM of the primary antibody incubated with 9.5 nM Saporin-Ab (Fig. 2C). Then, we evaluated cytotoxicity of immunotoxin on B16 melanoma cells (GM3^+^) genetically modified to express the ganglioside GD3 by stable transfection with Sial-T2. The expression of GD3 was clearly observed at the plasma membrane of live cells a 4°C, which was latter endocytosed by changing the temperature to 37°C (Fig. 3A). In contraposition to CHO-K1^GD3+^ and SK-Mel-28 cells, GD3-R24 did not significantly colocalyze with coendocytosed transferrin in experimental conditions set up to label recycling endosomes (30 min of uptake at 37°C). However, we observed extensive colocalization with Rab5 (early endosome marker) and lysosome-associated membrane protein 1 (Lamp1) (late endosome and lysosome marker), indicating that the R24 antibody is probably targeted to the degradation pathway (Fig. 3B). Independent of these alternative intracellular routes, we were also able to demonstrate selective cytotoxicity of immunotoxin on mouse B16^(GD3+)^ melanoma cells (Fig. 3C). Taken together, our results indicate that the ganglioside GD3 is a valid target for antibody-mediated intracellular delivery of cytotoxic agents in different cell types.

### Selective Delivery of Toxin Via R24 Antibody Drastically Inhibit the Clonogenic Growth of GD3-Expressing Cells

**Figure 4:**
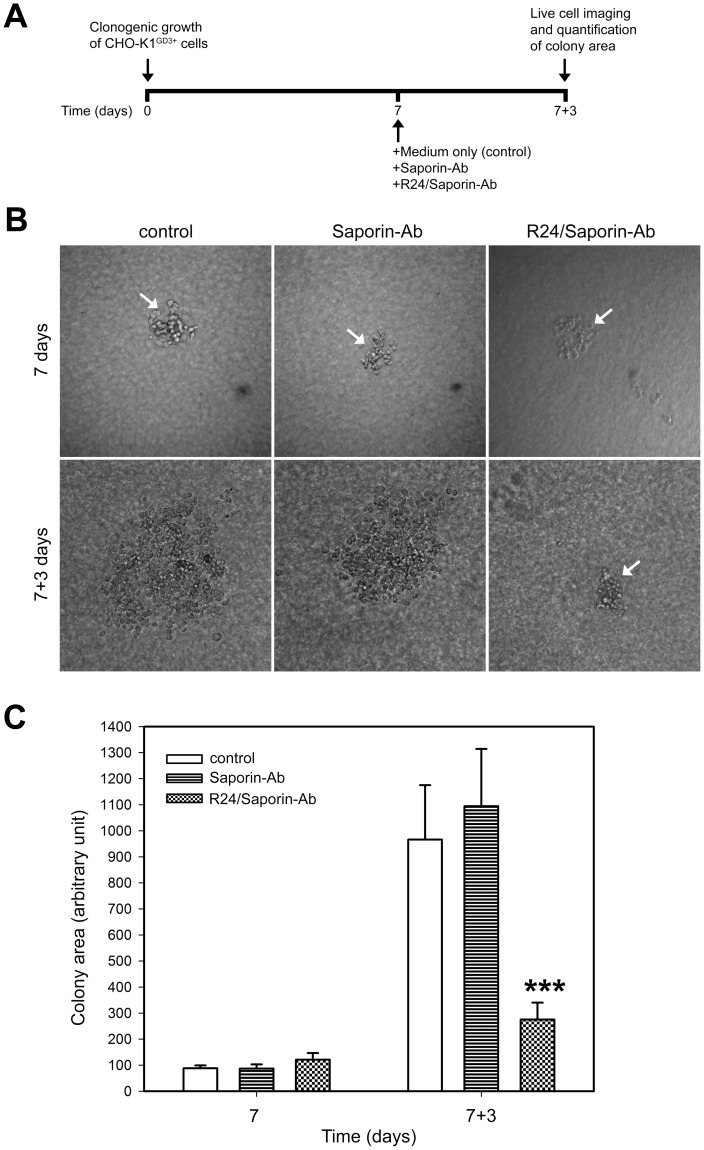
Targeted delivery of immunotoxin by R24 antibody inhibit the clonogenic growth of CHO-K1^GD3+^ cells. A ) A schematical representation of the experimental procedure used in (**B** and **C**). **B**) CHO-K1^GD3+^ cells (50–80 cells) were grown in 24-well plates previously coated with 0.5% agar in DMEM supplemented with 20% FBS. Cells were maintained at 37°C in a hummed atmosphere until cell colonies appeared (7 days, upper row. Colonies indicated with arrows). Then, cells were treated for 3 days (7+3 days, lower row) with 0.95 nM Saporin-Ab (Saporin-Ab, middle panel) or 30 nM R24/0.95 nM Saporin-Ab (R24/Saporin-Ab, right panel). CHO-K1^GD3+^ cells maintained only with medium were used as negative control (control, left panel). The micrographs are representative of three independent experiments. **C**) Quantification of the colony area at 7 and 7+3 days at the different conditions indicated in **B**. Results were analyzed by ANOVA followed by Tukey’s multiple comparison test. Results are given as means±S.E. Note that the clonogenic growth of CHO-K1^GD3+^ cells was severely affected only in presence of R24/Saporin-Ab (*** p<0.0001, respect to control condition at 7+3 days).

To estimate the potential antitumor activity of R24/Saporin-Ab complex, we evaluated the effect of the immunotoxin on the clonogenic growth of CHO-K1 cells grown on semisolid medium. CHO-K1 cells expressing the ganglioside GD3 were grown during 7 days to allow the formation of colonies containing approximately 60–80 cells. Then, cells were exposed to 30 nM R24/0.95 nM Saporin-Ab and the size of the colony scored at different times. As shown in Figure 4B and C, a drastic growth inhibition of CHO-K1^GD3+^ cells was reached after 3 days of immunotoxin treatment. By the contrary, CHO-K1^GD3+^ colonies continue to growth at the same concentration of the immuntoxin, but in the absence of R24 antibody, or in the absence of both immunotoxin and R24, undoubtedly indicating the specificity of the effect observed.

**Figure 5:**
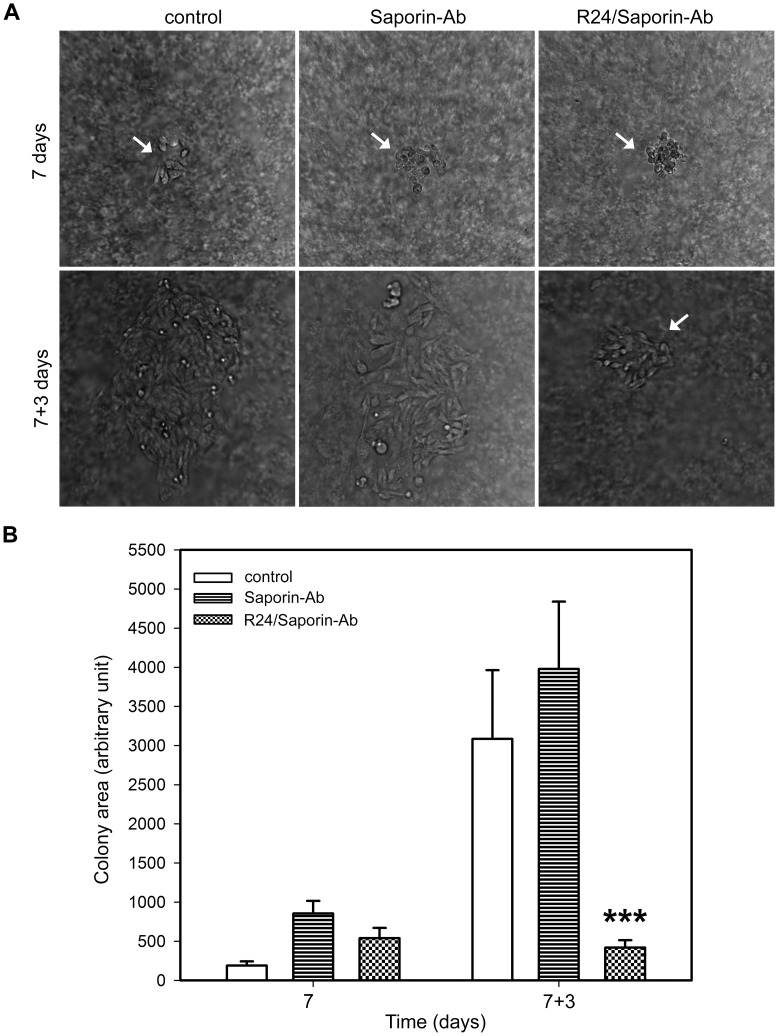
Selective delivery of saporin via R24 antibody drastically reduces the clonogenic growth of human SK-Mel-28 melanoma cells. A) SK-Mel-28 cells (50–80 cells) were grown in 24-well plates previously coated with 0.5% agar in DMEM supplemented with 20% FBS. Cells were maintained at 37°C in a hummed atmosphere until cell colonies appeared (7 days). Then, cells were exposed for 3 days (7+3 days) to 0.95 nM Saporin-Ab or 30 nM R24/0.95 nM Saporin-Ab. Colonies are indicated with arrows. **B)** Quantification of the colony area was performed at 7 and 7+3 days. SK-Mel-28 cells maintained only with medium were used as negative control (control). Results were analyzed by ANOVA followed by Tukey’s multiple comparison test. Results are given as means±S.E. Note that the clonogenic growth of SK-Mel-28 cells was severely affected only in presence of R24/Saporin-Ab (***p<0.0001, respect to control condition at 7+3 days).

**Figure 6:**
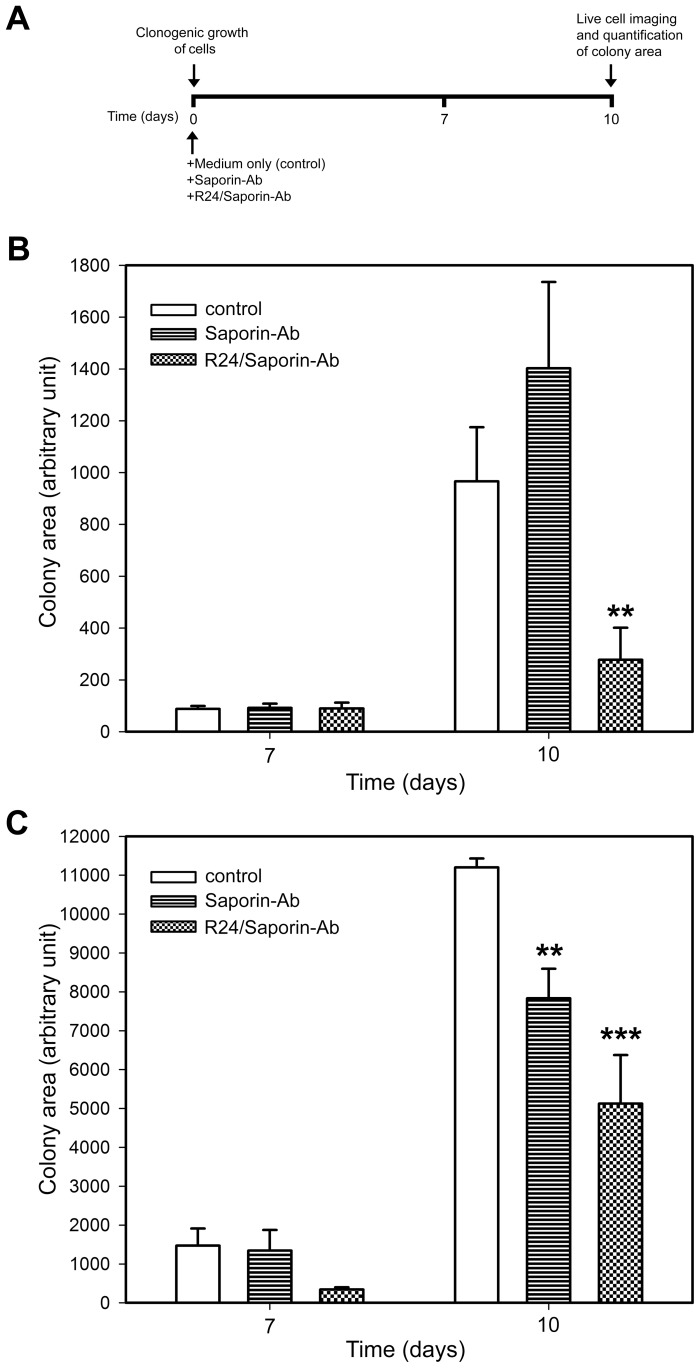
Immunotoxin inhibits CHO-K1^GD3+^ and SK-Mel-28 cells colony formation. A ) A schematical representation of the experimental procedure used in **B** and **C**. CHO-K1^GD3+^(**B**) and SK-Mel-28 (**C**) cells (50–80 cells) were seed in 24-well plates previously coated with 0.5% agar in DMEM supplemented with 20% FBS. Cultures were supplemented with 0.95 nM Saporin-Ab or 30 nM R24/0.95 nM Saporin-Ab and maintained at 37°C in a hummed atmosphere. Quantification of the colony area was performed every day, but only indicated at 7 and 10 days. Cells maintained only with medium were used as negative control (control). Results were analyzed by ANOVA followed by Tukey’s multiple comparison test. Results are given as means±S.E. Note the drastic inhibition of colony formation only in presence of R24/Saporin-Ab.

Next, we investigated the effect of the immunotoxin on the clonogenic growth of human SK-Mel-28 melanoma cell. As observed in CHO-K1 cells, the complex R24/Saporin-Ab had a significant and specific effect on the growing of the colonies (Fig. 5A and B). Human SK-Mel-28 melanoma cells grown in attachment-free conditions were more sensible to the action of saporin than in 2D monolayer. The concentration of saporin-Ab used for this cell line in clonogenic assays was 10-fold lower than in the 2D monolayer experiments. Interestingly, a drastic effect of the immunotoxin on the clonogenic growth of CHO-K1^GD3+^ and SK-Mel-28 cells was also observed when the cells were treated with the complex R24/Saporin-Ab at the moment of plating the cells in the semisolid medium, before colony formation (Fig. 6B and C). Taken together, these results clearly indicated that the complex R24/Saporin-Ab exert a notable cytotoxic effect on tumor cells grew both on 2D monolayers or cultured in attachment-free conditions, being more evident in the last culture condition.

## Discussion

The use of antibodies to specifically target different cell populations has become an increasingly desirable method for treatment of a variety of diseases. Cell surface receptors are main targets for immunotherapy due to the fact that they often play important roles in tumor biology, where they are overexpressed or display abnormal signaling. Therapeutic strategies include the use of *i*) unlabeled antibodies that kill tumor cells through induction of apoptosis, antibody-dependent cell-mediated cytotoxicity, and/or complement-dependent cytotoxicity; *ii*) radiolabeled antibodies to induce response in patients who are resistant to unlabeled antibodies and *iii*) antibody-drug conjugates, wherein a highly potent cytotoxic agent (drugs or toxins) is directed to the tumor by appending it to an antibody. In the last strategy, the cytotoxic agent is released upon internalization of the antibody-drug complex and causes the cell death.

The disialo ganglioside GD3 is a glycolipid highly expressed at early developmental stages of the central nerve system, when neural cells proliferate actively. At later developmental stages, the GD3 content declines and others gangliosides become major species [12], [13]. In addition, the expression level of gangliosides in general, and GD3 in particular, is very low and restricted in adult extra neural tissues. However, GD3 is highly expressed in tumor cells, accounting for more than 80% of melanomas. It is also overexpressed in neuroectodermal tumors (neuroblastoma and glioma) and carcinomas, including lung, breast, colon, prostate and ovary [14]. For these reasons, ganglioside GD3 has received considerable attention as a promising immunotherapeutic target for cancer therapy. As such, it has been used for passive [15] and active [16] immunotherapy of melanoma cancer. However, results with antibody therapy are still modest and generation of new GD3-specific chimeric antigen receptors with improved efficacy in human primary T lymphocytes is being evaluated [14].

We demonstrated that the R24 antibody to GD3 is rapidly endocytosed after binding to the disialo ganglioside at the cell surface, sorted to early endosomes and latter accumulated in recycling endosome [8]. Here, we also demonstrated in B16^GD3+^ melanoma cells that R24 antibody follows an alternative endocytic route, being probably targeted to the lysosomal degradation pathway. In this work, we exploited the internalization feature of the R24 antibody for the selective delivery of the cytotoxic agent saporin to GD3-expressing cells. Saporin is found in seeds and leaves of the plant *Saponaria officinales* and function as an RNA N-glycosidase and inhibits protein synthesis by cleaving one specific adenine base from ribosomal RNA and inducing irreversible ribosomal damage [17]. Saporin does not appear to possess putative translocation domain(s). Thus, the precise mechanism(s) used by saporin to reach the cytosolic compartment remains still unclear.

We had previously demonstrated that GD3 was suitable to mediate the intracellular delivery of R24 antibody alone or associated with a secondary anti mouse IgG antibody-Alexa Fluor^488^ [8]. Thus, we took advantage of this internalization feature of R24 antibody for selective delivery of saporin using a goat anti mouse IgG antibody linked to the ribosome-inactivating toxin. The immunotoxin was found to be specifically cytotoxic for GD3-expressing CHO-K1 and melanoma cells grown both on 2D monolayers and cultured in attachment-free conditions (clonogenic growth). We know that R24/saporin-Ab complex binds GD3 at the cells surface of CHO-K1 and SK-Mel-28 cells, being later endocytosed probably by a clathrin independent process [8]. After internalization, the immunotoxin complex transits the endosomal compartments and accumulates mainly in the recycling endosome, from where we speculate saporin translocate to the cytosolic compartment. At the cytosol, saporin inhibits protein synthesis and induces apoptosis.

Interestingly, biotinylated R24 antibody was also effective for targeted delivery of streptavidin-saporin, resulting in a significant reduction of viability of GD3-expressing cells (Figure S3). This result indicates that a simplified and safer R24-immunotoxin complex might be used in future evaluation of its potential therapeutic, precluding antigen competition between the saporin-conjugated secondary antibody with immunoglobulin from the host.

Antibody-drug conjugates are emerging as a highly effective therapy for cancer. Recent developments have led to an increase in the number of antibody-drug conjugates being tested clinically [18]. From our study, the ganglioside GD3 emerges as a novel and attractive class of cell surface molecule (glycolipid) for targeted delivery of drugs. The reason for its attractiveness is its accessibility, low expression on normal cells, high expression in many tumor cells, mainly those from neuroectodermal and epithelial origin, and mainly for its capacity to undergo endocytosis after binding with extracellular ligands such as antibodies.

In conclusion, results from this study point out that, independently of the endocytic pathway followed by the R24 antibody, it is possible to potentiate its cytotoxic properties on target cells by linking it to saporin or, eventually, to other drugs such as paclitaxel (Taxol) and doxorubicin [19], [20]. We hypothesize that this will aid in bypassing multiple drug resistance mediated by the p-glycoprotein pumps. Furthermore, a synergistic cytotoxic effect of R24 antibody-drug conjugate could be achievable by combination with antineoplastic agents, as previously reported [21]. Finally, other roles for R24 antibody could be envisaged: it could be used to develop nanoparticles for active drug targeting of nanomedicines, which eventually could also carrier fluorescent molecules for imaging cancer cells.

## Supporting Information

Figure S1 **Analysis of the intracellular fate of R24 alone or coupled to Saporin-Ab.** CHO-K1^GD3+^ cells grown on coverslips were incubated at 4°C to inhibit intracellular transport, then with R24 antibody or R24/Saporin-Ab for 45 min at 4°C, washed and fixed (A) or after washing the temperature was shifted to 37°C for 30 min to allow the endocytosis, washed and fixed (B). R24 antibody was detected by using goat anti-mouse IgG conjugated with Alexa Fluor^488^ (upper panels). R24/Saporin-Ab was detected by using rabbit anti-goat IgG conjugated with Alexa Fluor^488^ (lower panels). Single confocal sections were taken every 0.8 µm parallel to the coverslip. Scale bar: 10 µm.
(TIF)

Figure S2 **GD3 expression after prolonged R24-saporin-Ab treatment.** CHO-K1^GD3+^ cells were cultured at 37°C for 72 h in 96-well plates and treated with 20 nM monoclonal antibody to GD3 R24 (R24) in combination with secondary antibody (0.95 nM): goat antibody to mouse IgG (R24/anti-mouse IgG) or saporin conjugated goat antibody to mouse IgG (R24/Saporin-Ab). Then, cells were seed on coverslips, fixed and incubated with R24 antibody. The primary antibody was detected by using goat anti-mouse IgG conjugated with Alexa Fluor^488^. Single confocal sections were taken every 0.8 µm parallel to the coverslip. Scale bar: 10 µm.
(TIF)

Figure S3 **Selective cytotoxicity of R24-biotin/streptavidin-saporin on GD3 expressing cells. A**) Different amounts of R24 or R24-biotin (1 and 3, 0.4 µg; 2 and 4, 0.8 µg) were subjected to Western blot, stained with streptavidin (IRDye 680) and antibody (Ab) to mouse IgG (IRDye 800) and simultaneously detected using the Li-COR imaging system (Li-COR Biotechnology, Lincoln, NE, USA). **B**) CHO-K1^GD3+^ and SK-Mel-28 cells grown on coverslips were incubated at 4°C to inhibit intracellular transport, then with R24-biotin antibody for 45 min at 4°C, washed and fixed. R24-biotin was detected by using anti-mouse IgG conjugated with Alexa Fluor^488^. Single confocal sections were taken every 0.8 µm parallel to the coverslip. The fluorescence micrographs shown are representative of three independent experiments. Scale bar: 10 µm. **C**) SK-Mel-28 cells were cultured at 37°C for 72 h in 96-well plates and treated with or without R24-biotin in combination with antibody (Ab) to mouse IgG (0,78 nM) or streptavidin-saporin (0,78 nM, Advance Targeting Systems, San Diego, CA, USA). As control (100% viability), SK-Mel 28 cells were incubated only with culture medium. Cell viability was determined using the colorimetric MTT metabolic activity assay. Absorbance was measured at 595 nm using a multiplate reader. Results were analyzed by ANOVA followed by Tukey’s multiple comparison test. Results are three as means±S.E. The relative cell viability (%) was expressed as a percentage relative to the untreated control cells. Note that R24-biotin/streptavidin-saporin complex shows selective and specific cytotoxicity on melanoma cells (*, respect to control condition).
(TIF)

## References

[1] Daniotti JL, Iglesias-Bartolome R (2011) Metabolic pathways and intracellular trafficking of gangliosides. IUBMB Life 63: 513–520. 21698755 10.1002/iub.477

[2] Hakomori S (2002) Glycosylation defining cancer malignancy: new wine in an old bottle. Proc Natl Acad Sci U S A 99: 10231–10233. 12149519 10.1073/pnas.172380699 PMC124893

[3] Lee FT, Rigopoulos A, Hall C, Clarke K, Cody SH, et al (2001) Specific localization, gamma camera imaging, and intracellular trafficking of radiolabelled chimeric anti-G(D3) ganglioside monoclonal antibody KM871 in SK-MEL-28 melanoma xenografts. Cancer Res 61: 4474–4482. 11389078

[4] Schengrund CL, Shochat SJ (1988) Gangliosides in neuroblastomas. Neurochem Pathol 8: 189–202. 3075728 10.1007/BF03160146

[5] Allen TM (2002) Ligand-targeted therapeutics in anticancer therapy. Nat Rev Cancer 2: 750–763. 12360278 10.1038/nrc903

[6] Thomas PB, Delatte SJ, Sutphin A, Frankel AE, Tagge EP (2002) Effective targeted cytotoxicity of neuroblastoma cells. J Pediatr Surg 37: 539–544. 11877684 10.1053/jpsu.2002.30856

[7] Pukel CS, Lloyd KO, Travassos LR, Dippold WG, Oettgen HF, et al (1982) GD3, a prominent ganglioside of human melanoma. Detection and characterisation by mouse monoclonal antibody. J Exp Med 155: 1133–1147. 7061953 10.1084/jem.155.4.1133 PMC2186649

[8] Iglesias-Bartolome R, Crespo PM, Gomez GA, Daniotti JL (2006) The antibody to GD3 ganglioside, R24, is rapidly endocytosed and recycled to the plasma membrane via the endocytic recycling compartment. Inhibitory effect of brefeldin A and monensin. Febs J 273: 1744–1758. 16623710 10.1111/j.1742-4658.2006.05194.x

[9] Crespo PM, Zurita AR, Daniotti JL (2002) Effect of gangliosides on the distribution of a glycosylphosphatidylinositol-anchored protein in plasma membrane from Chinese hamster ovary-K1 cells. J Biol Chem 277: 44731–44739. 12237294 10.1074/jbc.M204604200

[10] Daniotti JL, Martina JA, Giraudo CG, Zurita AR, Maccioni HJ (2000) GM3 alpha2,8-sialyltransferase (GD3 synthase): protein characterization and sub-golgi location in CHO-K1 cells. J Neurochem 74: 1711–1720. 10737630 10.1046/j.1471-4159.2000.0741711.x

[11] Daniotti JL, Zurita AR, Trindade VM, Maccioni HJ (2002) GD3 expression in CHO-K1 cells increases growth rate, induces morphological changes, and affects cell-substrate interactions. Neurochem Res 27: 1421–1429. 12512945 10.1023/a:1021684018665

[12] Daniotti JL, Rosales Fritz V, Kunda P, Nishi T, Maccioni HJ (1997) Cloning, characterization and developmental expression of alpha2,8 sialyltransferase (GD3 synthase, ST8Sia I) gene in chick brain and retina. Int J Dev Neurosci 15: 767–776. 9402227 10.1016/s0736-5748(97)00027-0

[13] Gravotta D, Landa CA, Panzetta P, Maccioni HJ (1989) In vivo and in vitro expression of gangliosides in chick retina Mueller cells. J Neurochem 52: 768–776. 2645382 10.1111/j.1471-4159.1989.tb02521.x

[14] Lo AS, Ma Q, Liu DL, Junghans RP (2010) Anti-GD3 chimeric sFv-CD28/T-cell receptor zeta designer T cells for treatment of metastatic melanoma and other neuroectodermal tumors. Clin Cancer Res 16: 2769–2780. 20460472 10.1158/1078-0432.CCR-10-0043

[15] Nasi ML, Meyers M, Livingston PO, Houghton AN, Chapman PB (1997) Anti-melanoma effects of R24, a monoclonal antibody against GD3 ganglioside. Melanoma Res 7 Suppl 2S155–162. 9578432

[16] Ravindranath MH, Morton DL (1991) Role of gangliosides in active immunotherapy with melanoma vaccine. Int Rev Immunol 7: 303–329. 1779175 10.3109/08830189109114877

[17] Stirpe F, Barbieri L, Battelli MG, Soria M, Lappi DA (1992) Ribosome-inactivating proteins from plants: present status and future prospects. Biotechnology (N Y) 10: 405–412. 1368484 10.1038/nbt0492-405

[18] Alley SC, Okeley NM, Senter PD (2010) Antibody-drug conjugates: targeted drug delivery for cancer. Curr Opin Chem Biol 14: 529–537. 20643572 10.1016/j.cbpa.2010.06.170

[19] Guillemard V, Saragovi HU (2001) Taxane-antibody conjugates afford potent cytotoxicity, enhanced solubility, and tumor target selectivity. Cancer Res 61: 694–699. 11212270

[20] Guillemard V, Uri Saragovi H (2004) Prodrug chemotherapeutics bypass p-glycoprotein resistance and kill tumors in vivo with high efficacy and target-dependent selectivity. Oncogene 23: 3613–3621. 15034547 10.1038/sj.onc.1207463

[21] Polito L, Bolognesi A, Tazzari PL, Farini V, Lubelli C, et al (2004) The conjugate Rituximab/saporin-S6 completely inhibits clonogenic growth of CD20-expressing cells and produces a synergistic toxic effect with Fludarabine. Leukemia 18: 1215–1222. 15103391 10.1038/sj.leu.2403378

