# Supplementary figures and images for: Targeted Delivery of Immunotoxin by Antibody to Ganglioside GD3: A Novel Drug Delivery Route for Tumor Cells
Source: PLoS One. 2013 Jan 31;8(1):e55304. doi: 10.1371/journal.pone.0055304 (PMC3561269; doi:10.1371/journal.pone.0055304)

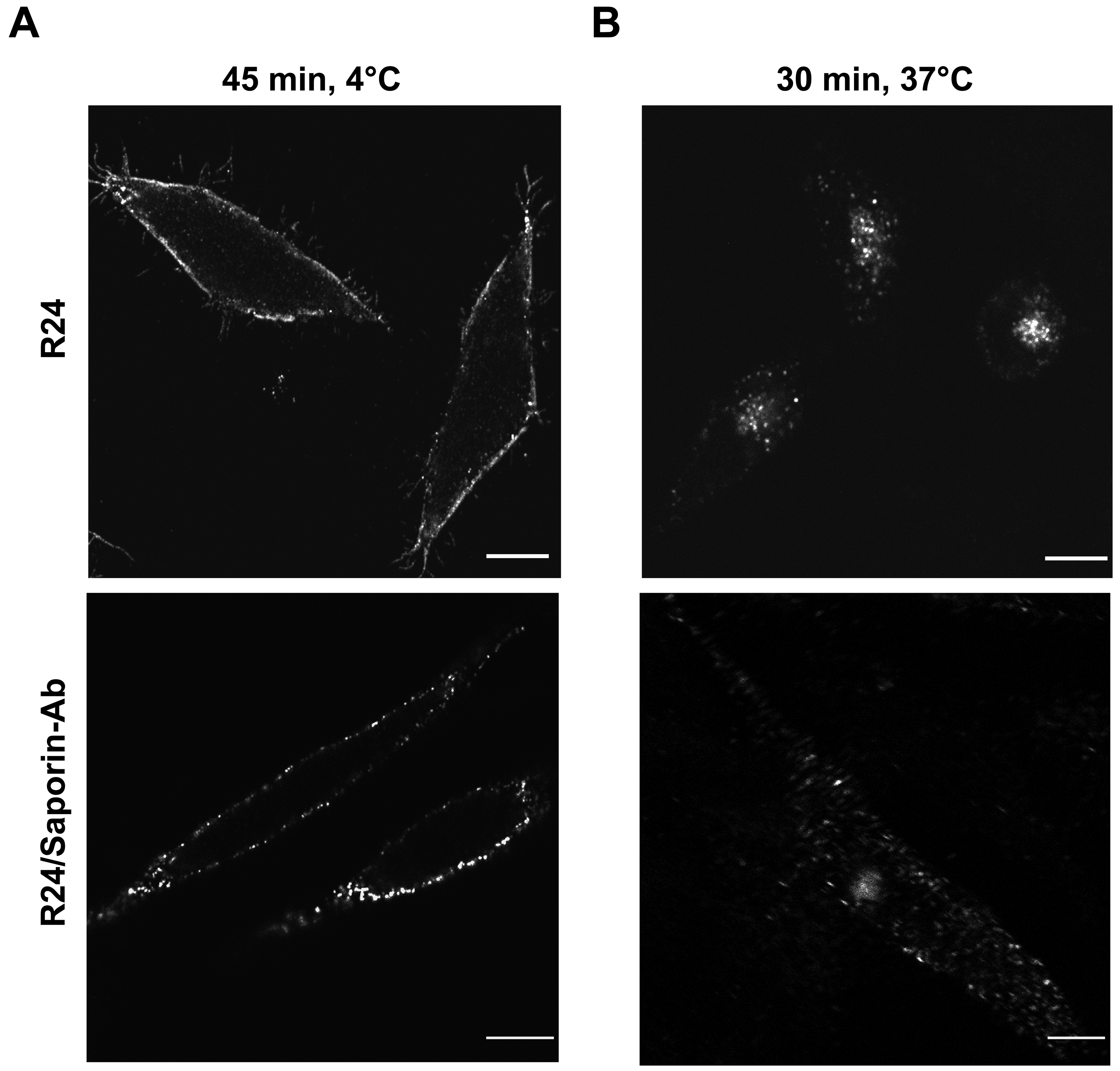

Supplement: Figure S1 — Analysis of the intracellular fate of R24 alone or coupled to Saporin-Ab. CHO-K1GD3+ cells grown on coverslips were incubated at 4°C to inhibit intracellular transport, then with R24 antibody or R24/Saporin-Ab for 45 min at 4°C, washed and fixed (A) or after washing the temperature was shifted to 37°C for 30 min to allow the endocytosis, washed and fixed (B). R24 antibody was detected by using goat anti-mouse IgG conjugated with Alexa Fluor488 (upper panels). R24/Saporin-Ab was detected by using rabbit anti-goat IgG conjugated with Alexa Fluor488 (lower panels). Single confocal sections were taken every 0.8 µm parallel to the coverslip. Scale bar: 10 µm. (TIF) [file pone.0055304.s001.tif]

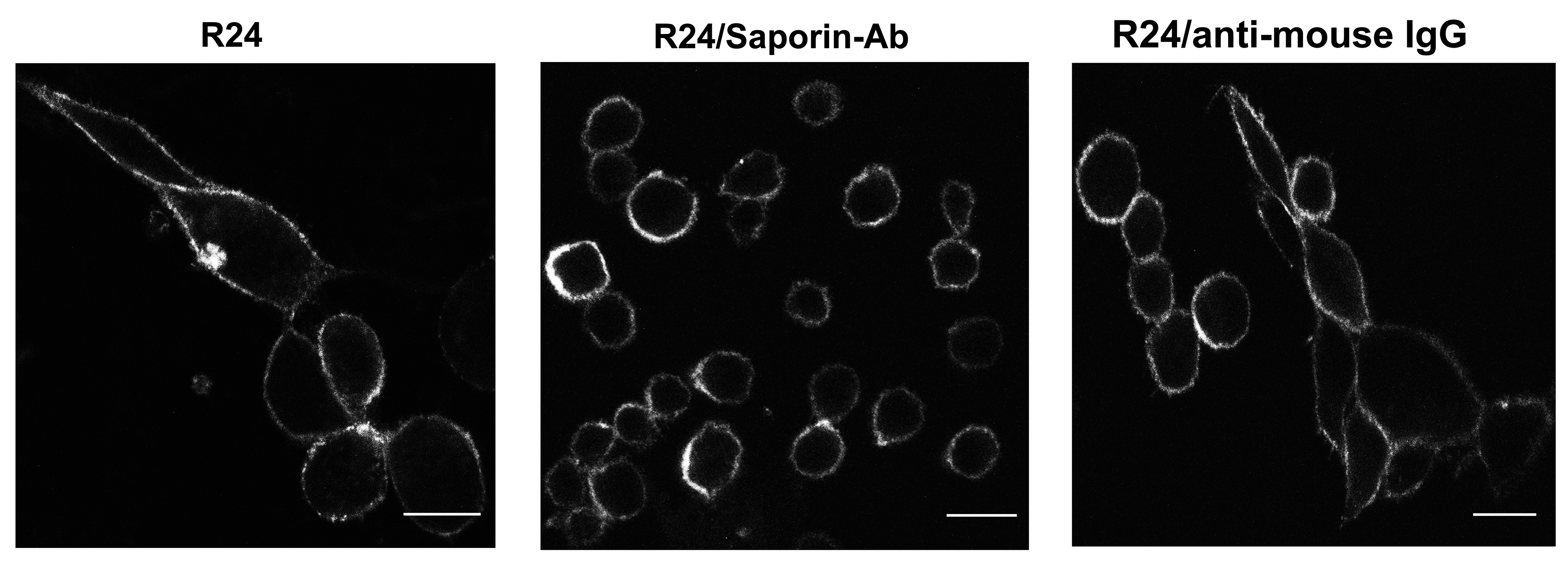

Supplement: Figure S2 — GD3 expression after prolonged R24-saporin-Ab treatment. CHO-K1GD3+ cells were cultured at 37°C for 72 h in 96-well plates and treated with 20 nM monoclonal antibody to GD3 R24 (R24) in combination with secondary antibody (0.95 nM): goat antibody to mouse IgG (R24/anti-mouse IgG) or saporin conjugated goat antibody to mouse IgG (R24/Saporin-Ab). Then, cells were seed on coverslips, fixed and incubated with R24 antibody. The primary antibody was detected by using goat anti-mouse IgG conjugated with Alexa Fluor488. Single confocal sections were taken every 0.8 µm parallel to the coverslip. Scale bar: 10 µm. (TIF) [file pone.0055304.s002.tif]

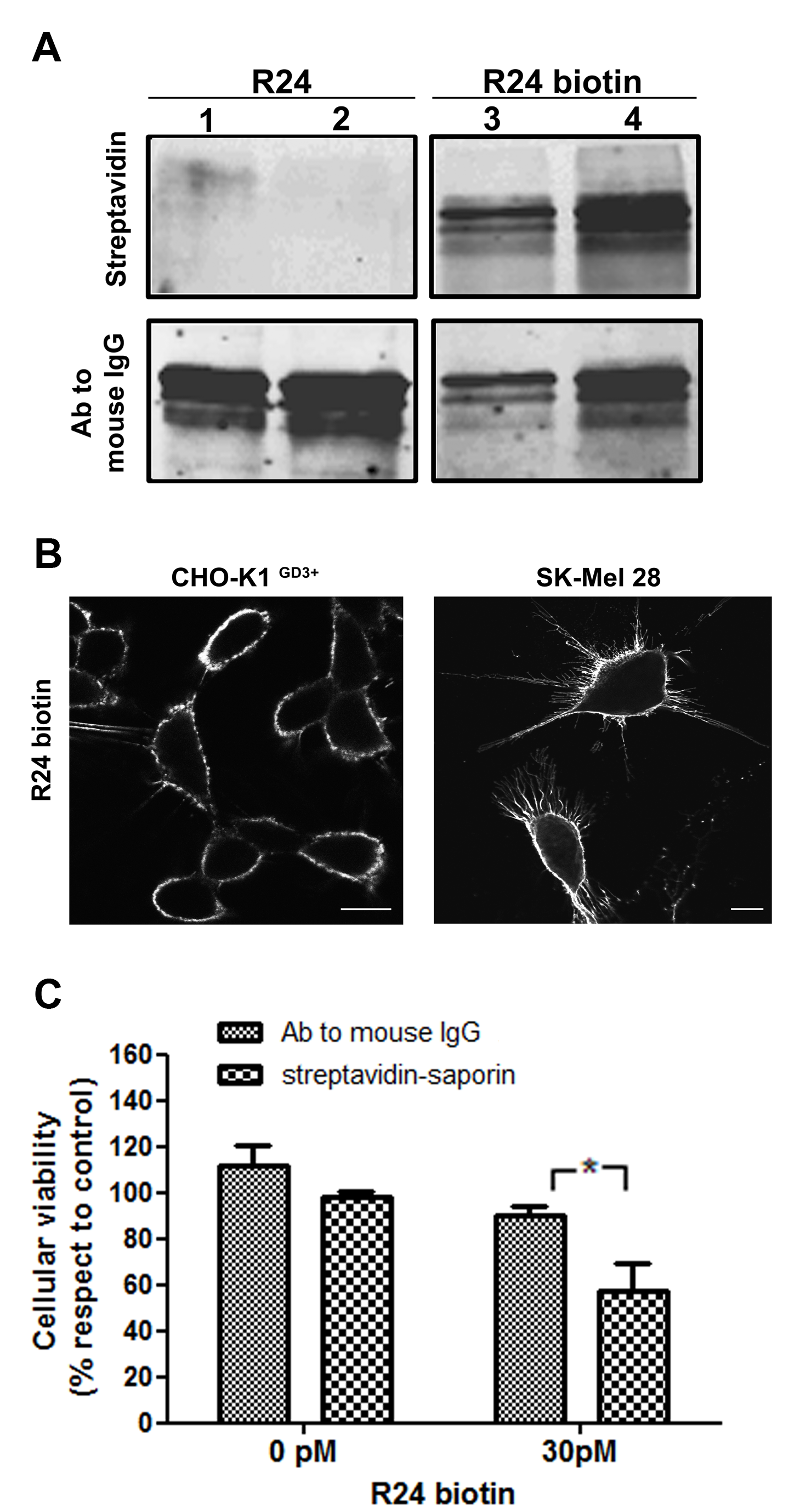

Supplement: Figure S3 — Selective cytotoxicity of R24-biotin/streptavidin-saporin on GD3 expressing cells. A) Different amounts of R24 or R24-biotin (1 and 3, 0.4 µg; 2 and 4, 0.8 µg) were subjected to Western blot, stained with streptavidin (IRDye 680) and antibody (Ab) to mouse IgG (IRDye 800) and simultaneously detected using the Li-COR imaging system (Li-COR Biotechnology, Lincoln, NE, USA). B) CHO-K1GD3+ and SK-Mel-28 cells grown on coverslips were incubated at 4°C to inhibit intracellular transport, then with R24-biotin antibody for 45 min at 4°C, washed and fixed. R24-biotin was detected by using anti-mouse IgG conjugated with Alexa Fluor488. Single confocal sections were taken every 0.8 µm parallel to the coverslip. The fluorescence micrographs shown are representative of three independent experiments. Scale bar: 10 µm. C) SK-Mel-28 cells were cultured at 37°C for 72 h in 96-well plates and treated with or without R24-biotin in combination with antibody (Ab) to mouse IgG (0,78 nM) or streptavidin-saporin (0,78 nM, Advance Targeting Systems, San Diego, CA, USA). As control (100% viability), SK-Mel 28 cells were incubated only with culture medium. Cell viability was determined using the colorimetric MTT metabolic activity assay. Absorbance was measured at 595 nm using a multiplate reader. Results were analyzed by ANOVA followed by Tukey’s multiple comparison test. Results are three as means±S.E. The relative cell viability (%) was expressed as a percentage relative to the untreated control cells. Note that R24-biotin/streptavidin-saporin complex shows selective and specific cytotoxicity on melanoma cells (*, respect to control condition). (TIF) [file pone.0055304.s003.tif]
